# Co-Expression of PA0290 and PelD Enhances *Pseudomonas aeruginosa* Biofilm Formation

**DOI:** 10.3390/microorganisms14091961

**Published:** 2026-09-05

**Authors:** Xueliang Zhan, Yushan Pei, Yuanyuan Huang, Na Li, Wenhui Huang, Yitong Tang, Haihua Liang

**Affiliations:** 1 Department of Clinical Laboratory, Shenzhen Third People’s Hospital (Second Affiliated Hospital of Southern University of Science and Technology), National Clinical Research Center for Infectious Diseases, Shenzhen 518112, China; zxl122012@gmail.com; 2 School of Medicine, Southern University of Science and Technology, Shenzhen 518055, China; 3 Medicine College, Hubei University of Arts and Science, Xiangyang 441000, China

**Keywords:** *Pseudomonas aeruginosa*, c-di-GMP, diguanylate cyclase, PA0290, PelD, biofilm

## Abstract

How individual diguanylate cyclases generate specific outputs within bacterial c-di-GMP networks remains unclear. Here, we characterized PA0290, a PAS-PAC-GGDEF protein of *Pseudomonas aeruginosa*, and examined its relationship with the c-di-GMP receptor PelD. Deletion or overexpression of PA0290 alone did not significantly affect biofilm formation. A bacterial adenylate cyclase two-hybrid screen identified PelD as a candidate PA0290-interacting protein, and co-expression of PA0290 and PelD markedly enhanced static and flow-cell biofilm formation. Purified PA0290 generated an HPLC product peak with a retention time closely matching that of the authentic c-di-GMP standard, whereas substitution of the conserved GGEEF motif with GGAAF reduced product formation and weakened the biofilm-enhancing phenotype observed upon PA0290–PelD co-expression. PA0290–PelD co-expression did not produce sustained activation of the bulk c-di-GMP-responsive *cdrA-lux* reporter. Clinical isolates also displayed heterogeneous biofilm-forming capacity and variable *PA0290* and *pelD* transcript abundance. Together, these findings support a functional association between PA0290 and PelD in biofilm regulation. The absence of sustained bulk c-di-GMP-responsive reporter activation suggests that this phenotype is not accompanied by a generalized increase in c-di-GMP-responsive transcription.

## 1. Introduction

Biofilms are spatially organized microbial communities embedded in a self-produced matrix, but they can occur as surface-attached structures or suspended aggregates depending on the biological setting [1]. In *Pseudomonas aeruginosa*, biofilm growth contributes to persistent infection and markedly increases tolerance to antimicrobial treatment [2]. Recent single-cell imaging further showed that cellular arrangement within *P. aeruginosa* biofilms shapes metabolic activity and antibiotic susceptibility, emphasizing that total biomass alone does not capture biofilm physiology [3].

The second messenger cyclic diguanylate (c-di-GMP) coordinates the transition between motile and biofilm-associated lifestyles through the opposing activities of GGDEF-domain diguanylate cyclases and EAL- or HD-GYP-domain phosphodiesterases [4]. c-di-GMP also integrates nutrient limitation, oxidative stress, and other environmental challenges into adaptive physiological responses [5]. *P. aeruginosa* encodes an unusually large c-di-GMP network; a systematic phylogenetic and transcriptomic analysis identified PA0290 as a conserved GGDEF-domain protein and provided initial evidence of diguanylate cyclase activity [6]. PA0290 was also included in a comprehensive phenotypic analysis of *P. aeruginosa* mutants lacking c-di-GMP-related genes, further supporting the need to define its condition-specific physiological role. More recently, multiplex disruption of the GGDEF-protein repertoire highlighted extensive functional overlap and robustness within this network [7]. Thus, the major unresolved question for PA0290 is not whether it can synthesize c-di-GMP, but how its activity is connected to a specific physiological output.

Current models distinguish global c-di-GMP regulation from local signaling, in which a metabolic enzyme communicates preferentially with a nearby receptor or target [8]. Experimental support for local signaling is strongest when a specific phenotype, limited alteration of the bulk c-di-GMP pool, and physical association of pathway components are observed together [9]. In *P. aeruginosa*, the partner protein SiaC directly activates the diguanylate cyclase SiaD by stabilizing a catalytically competent assembly [10]. An iron-responsive IsmP–ImcA module similarly links an extracellular cue to regulated c-di-GMP synthesis and biofilm formation [11]. The diguanylate cyclase YfiN provides another example in which an environmental stress, peroxide exposure, is coupled to biofilm maintenance [12]. Environmental purines can also reprogram *P. aeruginosa* biofilm formation by altering c-di-GMP metabolism upstream of matrix production [13]. At the structural level, sensory-domain rearrangements can position GGDEF domains for productive catalysis, providing a mechanistic basis for stimulus-dependent diguanylate cyclase activation [14].

Pel is one of the principal exopolysaccharides contributing to *P. aeruginosa* biofilm architecture, and its synthesis, modification, and export are coordinated by the Pel machinery [15]. PelD is an inner-membrane c-di-GMP receptor required for efficient Pel-dependent exopolysaccharide production [16]. Structural analysis showed that its cytoplasmic region contains a degenerate GGDEF-like receptor module rather than an active catalytic center [17]. Pel can provide structural and protective functions within the biofilm matrix [18]. Its positive charge also enables cross-linking with extracellular DNA [19]. Recent work on BifA further demonstrated that PelD-associated signaling can influence Pel-dependent biofilm formation [20]. Despite this progress, the upstream diguanylate cyclases that preferentially communicate with PelD remain incompletely defined.

Here, we examined the functional relationship between PA0290 and PelD. Altering PA0290 abundance alone did not produce a statistically detectable biofilm phenotype under the conditions tested, whereas co-expression of PA0290 and PelD markedly enhanced biofilm formation. BACTH and affinity co-purification assays supported an association between the two proteins, while biochemical analysis supported diguanylate cyclase activity of PA0290. Substitution of the conserved GGEEF motif reduced both product formation in vitro and the biofilm-enhancing phenotype associated with PA0290–PelD co-expression. We further examined bulk c-di-GMP-responsive reporter activity and the relationships between *PA0290*/*pelD* expression and biofilm formation in clinical isolates.

## 2. Materials and Methods

### 2.1. Bacterial Strains, Plasmids and Growth Conditions

The bacterial strains and plasmids used in this study are listed in Table S1. *Pseudomonas aeruginosa* PAO1 and its derivatives were routinely cultured in LB broth at 37 °C with shaking at 220 rpm, unless otherwise indicated. *Escherichia coli* strains used for plasmid construction, protein expression and bacterial two-hybrid assays were cultured in LB medium at 37 °C. When required, antibiotics were added at the appropriate concentrations for plasmid maintenance and strain selection.

The PA0290-overexpression plasmid was generated by cloning the coding sequence of PA0290 into the compatible expression vector pME-6032. The *pelD* coding sequence was cloned into pAK-1900 to allow simultaneous expression of PA0290 and PelD in PAO1. For comparisons involving the two compatible expression plasmids, strains lacking a gene insert carried the corresponding empty vector so that vector backbones, antibiotic selection, and induction conditions were matched across groups.

### 2.2. Construction of the PA0290 Deletion, Complementation and Catalytic-Site Mutant Strains

A markerless in-frame deletion of PA0290 was generated using the pEX18Ap-based two-step allelic-exchange framework [21]. DNA fragments corresponding to the upstream and downstream regions of PA0290 were amplified from PAO1 genomic DNA, fused, and cloned into the suicide vector pEX18Ap. The recombinant plasmid was introduced into PAO1 by electroporation. Single-crossover recombinants were selected on antibiotic-containing agar, and double-crossover mutants were isolated by sucrose counterselection. The deletion was verified by diagnostic PCR and Sanger sequencing.

For genetic complementation, the intact PA0290 coding sequence was introduced into the ΔPA0290 mutant using pME-PA0290. To generate a catalytically impaired derivative, the conserved GGEEF motif of PA0290 was changed to GGAAF by site-directed mutagenesis. The resulting construct, designated PA0290^GGAAF^, was confirmed by Sanger sequencing.

### 2.3. Protein-Domain Analysis

The amino acid sequence of PA0290 was analyzed using SMART v10 to identify conserved protein domains [22]. PA0290 was predicted to contain an N-terminal PAS-PAC sensory region and a C-terminal GGDEF diguanylate cyclase domain. The domain architecture was illustrated using Adobe Illustrator.

### 2.4. Static Biofilm Assay

Static biofilm formation was assessed by crystal violet staining using a protocol adapted from the established microtiter-dish assay. Overnight bacterial cultures were diluted to an initial OD_600_ of 0.003 in fresh LB and inoculated into glass tubes at 2 mL per tube. Cultures were incubated without shaking at 37 °C for 16 or 24 h.

After incubation, planktonic cells were removed, and the attached biofilms were gently washed two times with phosphate-buffered saline (PBS). Biofilms were stained with 0.1% crystal violet for 20 min at room temperature. Excess stain was removed by washing with PBS, and the bound crystal violet was solubilized with 33% acetic acid. Absorbance was measured at 590 nm. Bacterial growth was determined at OD_600_, and biofilm formation was expressed as OD_590_/OD_600_.

The following strains were compared where applicable: PAO1/EV, ΔPA0290/EV, ΔPA0290/p-PA0290, PAO1/p-PA0290, PAO1/p-*pelD* and PAO1 co-expressing PA0290 and PelD. The effect of the GGEEF-to-GGAAF substitution on the PA0290–PelD-associated biofilm phenotype was examined by comparing PelD co-expression with wild-type PA0290 or PA0290^GGAAF^.

### 2.5. Bacterial Adenylate Cyclase Two-Hybrid Screening

Protein–protein interactions were screened using the bacterial adenylate cyclase two-hybrid (BACTH) system, which detects reconstitution of the T18 and T25 fragments of *Bordetella pertussis* adenylate cyclase [23]. The coding sequence of PA0290 was cloned into pUT18C to generate the pUT18C-PA0290 bait construct. Candidate genes encoding proteins associated with c-di-GMP metabolism and signaling were individually cloned into pKT25. For PelD (PA3061), the same cytoplasmic fragment used in the affinity co-purification assay was cloned for BACTH analysis. The corresponding pUT18C-PA0290 and pKT25-derived plasmids were co-transformed into *Escherichia coli* BTH101. Transformants were selected on LB agar containing 50 μg mL^−1^ ampicillin and 50 μg mL^−1^ kanamycin.

For qualitative interaction screening, individual transformants were spotted onto LB agar supplemented with 50 μg mL^−1^ ampicillin, 50 μg mL^−1^ kanamycin, 50 μg mL^−1^ X-gal and 0.5 mM IPTG. Plates were incubated at 30 °C for 48 h. Blue colony development indicated reconstitution of adenylate cyclase activity and was interpreted as evidence of a potential protein–protein interaction. The pUT18C-Zip/pKT25-Zip pair was used as the positive control, whereas the corresponding empty-vector combination was used as the negative control.

Interaction strength was further quantified by measuring β-galactosidase activity. Overnight cultures were diluted into fresh LB medium containing ampicillin and kanamycin and incubated at 30 °C until the OD_600_ reached approximately 0.8. Cells were permeabilized using SDS and chloroform, and o-nitrophenyl-β-D-galactopyranoside was added as the chromogenic substrate. Reactions were terminated by addition of sodium carbonate, and absorbance was measured at 420 and 550 nm. β-Galactosidase activity was calculated and expressed in Miller units.

The positive-interaction screening cutoff was defined as the mean β-galactosidase activity of the negative-control group plus three standard deviations (mean + 3 SD). Under the conditions used here, this corresponded to 538.23 Miller units. Candidate combinations with mean activity above this cutoff were classified as BACTH-positive. Reciprocal BACTH analysis of PA0290 and PelD was subsequently performed by testing both T18/T25 fusion orientations together with the corresponding empty-vector controls.

### 2.6. Affinity Co-Purification of PA0290 and the PelD Cytoplasmic Region

His-tagged PA0290 and the untagged cytoplasmic region of PelD (PelD^cyto^) were co-expressed from a pETDuet dual-expression vector in *Escherichia coli* BL21(DE3). The coding sequences of PA0290 and PelD^cyto^ were inserted into the two independent expression cassettes of the pETDuet vector to enable simultaneous expression from the same plasmid. Corresponding constructs expressing His-PA0290 alone or PelD^cyto^ alone were used as controls.

Cells were harvested after induction and resuspended in lysis buffer containing 50 mM HEPES (pH 8.0), 200 mM NaCl, and 10 mM imidazole. Cells were disrupted by sonication, and insoluble material was removed by centrifugation. The clarified soluble lysate was loaded onto a Ni^2+^–NTA affinity column pre-equilibrated with the same buffer. After sample loading, the column was washed with 10 column volumes (CV) of wash buffer containing 50 mM HEPES (pH 8.0), 200 mM NaCl, and 10 mM imidazole to remove nonspecifically bound proteins. Bound proteins were subsequently eluted using elution buffer containing 50 mM HEPES (pH 8.0), 200 mM NaCl, and 300 mM imidazole. Three consecutive elution fractions of 1 CV each were collected. Input and elution fractions were analyzed by SDS–PAGE. The appearance of an additional band at the expected molecular mass of PelD^cyto^ in the His-PA0290 co-elution fraction was considered biochemical evidence consistent with an association between the two proteins under the tested conditions.

### 2.7. Flow-Cell Biofilm Cultivation and Confocal Microscopy

Flow-cell biofilms were established using a continuous-flow chamber system. Overnight cultures were diluted in fresh LB and introduced into the chambers. Cells were allowed to attach without flow for 30 min, after which fresh medium was supplied at a constant flow rate of 3 mL h^−1^. Separate independently inoculated flow-cell chambers were used for the 16 h and 24 h endpoints. Thus, measurements at the two time points represented independent endpoint experiments rather than repeated measurements of the same chamber.

Biofilms were stained with SYTO 9 at a final concentration of 2 μM and incubated in the dark for 20 min. Excess dye was removed by gentle washing with PBS or fresh medium. Z-stack images were acquired using a ZEISS LSM 980 (Carl Zeiss Microscopy GmbH, Jena, Germany) confocal laser scanning microscope equipped with a 20× objective, with a step size of 1.5 μm.

Three-dimensional biofilm structures were reconstructed using Imaris. Biofilm biomass was quantified from the confocal image stacks using Imaris 9.0.1 (Oxford Instruments, Abingdon, UK). At least three independent confocal fields were acquired from each biological replicate. Biomass values from these fields were averaged to generate a single value for each biological replicate; thus, individual imaging fields were not treated as independent replicates for statistical analysis. The same Imaris segmentation and intensity-threshold settings were applied to all images within the same experiment.

### 2.8. Measurement of c-di-GMP-Responsive Luminescence

Intracellular c-di-GMP-responsive activity was monitored using the *cdrA-lux* reporter. The cdrA promoter is widely used as a transcriptional readout of c-di-GMP-responsive signaling in *P. aeruginosa*. The reporter plasmid containing the cdrA promoter fused to the luxCDABE operon was introduced into the indicated PAO1 derivatives.

Overnight cultures were diluted into fresh medium and transferred to white, clear-bottom microplates. Cultures were incubated at 37 °C in a multimode plate reader (BioTek Instruments, Winooski, VT, USA), and OD_600_ and luminescence were recorded at regular intervals for 24 h. Luminescence was expressed as counts per second normalized to bacterial growth at OD_600_ (CPS/OD_600_).

The *cdrA*-lux reporter was used as an indirect readout of cellular c-di-GMP-responsive signaling. Because this assay measures promoter-dependent luminescence rather than c-di-GMP directly, the results were interpreted as bulk c-di-GMP-responsive reporter activity.

To quantify cumulative reporter activity over the monitoring period, the area under the curve (AUC) from 0 to 24 h was calculated separately for each independent biological replicate using the trapezoidal method with a baseline of Y = 0. The resulting AUC values were used for statistical comparisons among strains.

### 2.9. Expression and Purification of PA0290 and PA0290^GGAAF^

The full-length coding sequences of PA0290 and its GGAAF variant, in which the native GGEEF motif was replaced with GGAAF, were cloned into the pET-28a-SUMO expression vector and transformed into *Escherichia coli* BL21(DE3). Protein expression was induced with 0.25 mM IPTG at 16 °C for 18 h.

Cells were harvested, resuspended in lysis buffer A containing 200 mM NaCl, 2 mM MgCl_2_, and 50 mM HEPES (pH 8.0), and disrupted by sonication. Following centrifugation, the His–SUMO-tagged proteins were purified by Ni^2+^–NTA affinity chromatography and exchanged into the enzymatic reaction buffer by centrifugal ultrafiltration. The His–SUMO tags were not removed. Protein purity and concentration were determined by SDS–PAGE and the BCA assay, respectively.

### 2.10. In Vitro Diguanylate Cyclase Assay

The diguanylate cyclase activity of PA0290 was assessed using GTP as the substrate. Purified wild-type PA0290 or the PA0290^GGAAF^ variant was added to a reaction buffer containing 50 mM HEPES (pH 8.0), 200 mM NaCl, and 2 mM MgCl_2_. The final concentrations of PA0290 protein and GTP were 50 μM and 100 μM, respectively. Reactions were incubated at 37 °C for 8 h. For the heat-inactivated control, purified wild-type PA0290 was pre-incubated at 65 °C for 30 min before GTP addition. At the end of the enzymatic reaction, all samples were heated at 65 °C for 30 min to terminate catalysis. Precipitated proteins were removed by centrifugation, and the resulting supernatants were analyzed by reversed-phase HPLC using established principles for c-di-GMP separation and detection.

GTP and c-di-GMP standards were prepared at 100 μM and analyzed under the same chromatographic conditions as the enzymatic reaction samples. Separation was performed on an Agilent HPLC system (Agilent Technologies, Santa Clara, CA, USA) equipped with an ACQUITY UPLC BEH C18 column (130 Å pore size, 1.7 μm particle size, 2.1 mm × 50 mm; Waters Corporation, Milford, MA, USA) maintained at room temperature. Mobile phase A consisted of 20 mM ammonium acetate in water, and mobile phase B consisted of 20 mM ammonium acetate in methanol. The flow rate was 0.25 mL min^−1^, and the injection volume was 5 μL. The gradient program was as follows: 1% B from 0 to 2 min; a linear increase from 1% to 30% B from 2 to 4 min; and an immediate return to 1% B at 4 min, followed by maintenance at 1% B until 10 min. GTP and c-di-GMP were monitored by ultraviolet absorbance at 254 nm. The reaction product was tentatively assigned as c-di-GMP based on retention-time matching with the authentic c-di-GMP standard. The catalytic activity of wild-type PA0290 was compared with those of PA0290^GGAAF^ and the heat-inactivated PA0290 control. Retention times and integrated peak areas are summarized in Table S4.

### 2.11. Clinical P. aeruginosa Isolates

Thirty-five non-duplicate clinical *Pseudomonas aeruginosa* isolates were obtained from an archived bacterial strain collection maintained by the clinical microbiology laboratory of The First Affiliated Hospital, Zhejiang University School of Medicine. These isolates had originally been recovered from routine diagnostic specimens between 2018 and 2023 and were retained after completion of the corresponding diagnostic procedures. The isolates were designated as non-duplicate by the source laboratory and were fully de-identified before being provided for this study. No patient identifiers, clinical records, or other personal information were accessed or analyzed.

### 2.12. Congo Red Colony-Morphology Assay

Colony morphology and extracellular matrix-associated pigmentation were examined on Congo red agar. Overnight cultures were normalized and spotted onto tryptone agar supplemented with Congo red at 40 µg mL^−1^ and Coomassie brilliant blue concentration at 20 µg mL^−1^. Plates were incubated at 25 °C for 48 h.

Colonies were photographed under identical illumination and exposure conditions. Differences in red pigmentation, colony rugosity and small-colony-like morphology were evaluated descriptively. Congo red morphology was used as a qualitative indicator of variation in extracellular matrix-associated colony phenotypes and was not used alone to define a genetically stable small-colony variant.

### 2.13. Biofilm Phenotyping of Clinical Isolates

Static biofilm formation by the 35 clinical isolates was measured using the crystal violet assay described above. Biofilm values were calculated as OD_590_/OD_600_. The resulting values were displayed as a heatmap to visualize inter-isolate variation in biofilm-forming capacity. Color intensity represented the measured value according to the corresponding color scale.

The heatmap was intended to illustrate the overall distribution and trend among isolates; no inferential statistical comparison was performed for the heatmap itself.

### 2.14. RNA Extraction and Quantitative Reverse-Transcription PCR

Clinical Pseudomonas aeruginosa isolates were cultured in LB broth at 37 °C with shaking at 220 rpm until the cultures reached an OD_600_ of approximately 1.0. Bacterial cells were harvested by centrifugation, and total RNA was extracted using the EasyPure^®^ RNA Kit (TransGen Biotech, Beijing, China; catalogue no. ER101-01) according to the manufacturer’s instructions. Residual genomic DNA was removed following the DNase treatment procedure provided with the kit. RNA concentration and purity were assessed using a NanoDrop spectrophotometer (Thermo Fisher Scientific, Waltham, MA, USA). Equal amounts of total RNA were reverse-transcribed into cDNA using the TransScript^®^ All-in-One First-Strand cDNA Synthesis SuperMix for qPCR (One-Step gDNA Removal) (TransGen Biotech, Beijing, China; catalogue no. AT341-01) according to the manufacturer’s protocol.

Quantitative reverse-transcription PCR was performed using SYBR Green chemistry on an Applied Biosystems 7500 Real-Time PCR System (Applied Biosystems, Foster City, CA, USA). Gene-specific primers were used to quantify PA0290 and *pelD* transcript levels, and rpoD served as the internal reference gene. Relative transcript abundance was calculated using the 2^−ΔΔCt^ method, with the designated reference isolate assigned a relative expression value of 1. PA0290 and *pelD* transcript levels were visualized as separate heatmaps using the same isolate order as that used for the biofilm-forming-capacity analysis. Color intensity represented relative transcript abundance. The heatmaps were used descriptively to visualize transcriptional variation among clinical isolates; no inferential statistical testing was applied to the heatmap color patterns. Primer efficiencies were determined from standard curves and were within the acceptable range for relative quantification. The amplification efficiencies of the PA0290, *pelD*, and *rpoD* primer pairs were 94.8%, 96.1%, and 95.3%, respectively, with corresponding R^2^ values of 0.991, 0.993, and 0.992. No-template and no-reverse-transcription controls were included to exclude reagent contamination and residual genomic DNA. The reference gene rpoD showed relatively limited Ct variation across the 35 isolates (overall mean ± SD, 18.52 ± 0.35; range, 17.85–19.18) and was therefore used for normalization. For each isolate, 3 biological replicates were analyzed, and each qRT-PCR reaction was performed in 3 technical replicates.

### 2.15. Identification of PA0290 and PelD Homologs and Phylogenetic Analysis

The PA0290 and PelD amino acid sequences from *P. aeruginosa* PAO1 were used as queries to search for homologous proteins in the NCBI non-redundant protein database using BLASTP v2.17.0. Homologs with ≥60% amino acid identity, ≥60% query coverage and an E-value ≤ 1 × 10^−5^ were retained.

Protein sequences were aligned using the MAFFT online service [24]. Poorly aligned regions were removed using trimAl. Maximum-likelihood phylogenetic trees were reconstructed with IQ-TREE 2. The best-fitting amino acid substitution model was selected using ModelFinder, and branch support was evaluated with 1000 UFBoot2 replicates. Trees were visualized and annotated using iTOL v6. Selected taxonomic groups were highlighted by colored sectors, and branch-length scale bars represent the estimated number of amino acid substitutions per site.

### 2.16. Statistical Analysis

Statistical analyses were performed using GraphPad Prism version 9.5. Data from independent biological experiments are presented as the mean ± standard deviation unless otherwise indicated.

Comparisons involving more than two groups at a single time point were analyzed using one-way ANOVA followed by Dunnett’s multiple-comparison test. Experiments involving both strain and incubation time were analyzed using two-way ANOVA followed by Šídák’s multiple-comparison test. For the *cdrA*-lux time-course experiment, the 0–24 h AUC was calculated separately for each independent biological replicate, and AUC values were compared using one-way ANOVA followed by Dunnett’s multiple-comparison test, with PAO1/EV as the reference group. β-Galactosidase activities were compared with the negative-control group using one-way ANOVA followed by Dunnett’s multiple-comparison test.

The clinical-isolate heatmaps of biofilm formation and gene expression were used descriptively to visualize inter-isolate trends, and no significance testing was applied to the heatmap color patterns themselves. Associations between biofilm formation and PA0290 or *pelD* transcript abundance across the 35 clinical isolates were assessed using Spearman rank correlation based on the mean value for each isolate. Ninety-five percent confidence intervals were calculated for Spearman’s correlation coefficients. To account for the two correlation tests, *p* values were adjusted using the Holm method. A Holm-adjusted *p* value < 0.05 was considered statistically significant.

## 3. Results

### 3.1. PA0290 Encodes a Putative Sensory Diguanylate Cyclase but Does Not Independently Regulate Biofilm Formation

The intracellular second messenger c-di-GMP is a central regulator of bacterial lifestyle transition, particularly the switch between motile growth and biofilm-associated growth in Pseudomonas aeruginosa. GGDEF domain-containing proteins constitute the major class of diguanylate cyclases responsible for c-di-GMP synthesis. However, increasing evidence indicates that many GGDEF proteins regulate specific cellular processes through spatially restricted signaling networks rather than simply altering the total intracellular c-di-GMP pool. Therefore, we first investigated the potential function of the previously uncharacterized GGDEF-containing protein PA0290.

**Figure 1:**
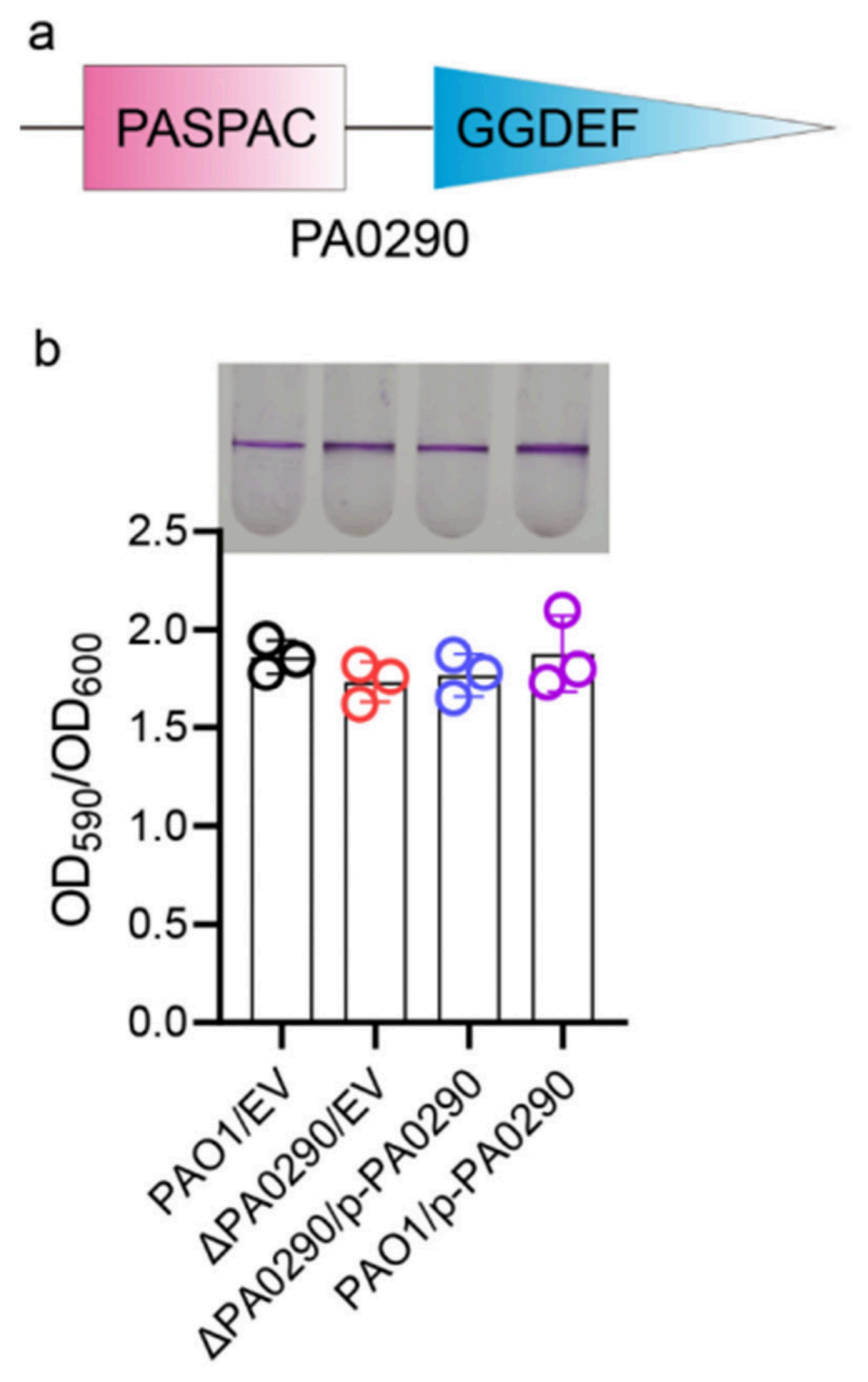
Domain architecture of PA0290 and its effect on static biofilm formation in *Pseudomonas aeruginosa*. (**a**) Schematic representation of the domain architecture of PA0290, which contains an N-terminal PAS-PAC sensory domain and a C-terminal GGDEF domain. (**b**) Static biofilm formation by PAO1 carrying the empty vector (PAO1/EV), the *PA0290* deletion mutant carrying the empty vector (Δ*PA0290*/EV), the complemented mutant (Δ*PA0290*/p-PA0290), and the PA0290-overexpression strain (PAO1/p-PA0290). Representative crystal violet-stained biofilms are shown above the quantitative results. Biofilm biomass was quantified by measuring crystal violet absorbance at 590 nm and normalized to bacterial growth at OD_600_. No significant differences in static biofilm formation were detected among the tested strains. Individual circles represent independent biological replicates, and bars indicate the mean ± SD (n = 3). EV, empty vector.

Bioinformatic analysis revealed that PA0290 comprises N-terminal PAS and PAC sensory domains and a C-terminal GGDEF-family domain harboring a GGEEF active-site motif (Figure 1a). This domain organization is characteristic of sensory diguanylate cyclases and suggested that PA0290 may participate in c-di-GMP signaling. To determine whether PA0290 contributes to biofilm development, we constructed a markerless PA0290 deletion mutant, its complemented derivative, and a PA0290-overexpression strain. Static biofilm formation was subsequently quantified using the crystal violet staining assay.

Compared with the wild-type PAO1 strain carrying the empty vector, deletion of PA0290 did not significantly affect biofilm biomass. Similarly, complementation of PA0290 deletion or overexpression of PA0290 failed to produce a significant increase in biofilm formation (Figure 1b). Under the conditions tested, neither deletion nor overexpression of PA0290 produced a statistically detectable change in static biofilm formation. These observations prompted us to examine whether PA0290 might exert a more evident phenotype in the presence of a specific interaction partner or downstream effector.

### 3.2. BACTH Screening Identifies PelD as a Candidate Interaction Partner of PA0290

Because PA0290 did not generate an obvious phenotype when expressed alone, we examined whether PA0290 might associate with other proteins involved in c-di-GMP metabolism or signaling. To identify potential partners involved in PA0290-mediated regulation, we performed a bacterial adenylate cyclase two-hybrid (BACTH) screen using PA0290 as bait against a panel of proteins associated with c-di-GMP metabolism and signaling in *P. aeruginosa*.

**Figure 2:**
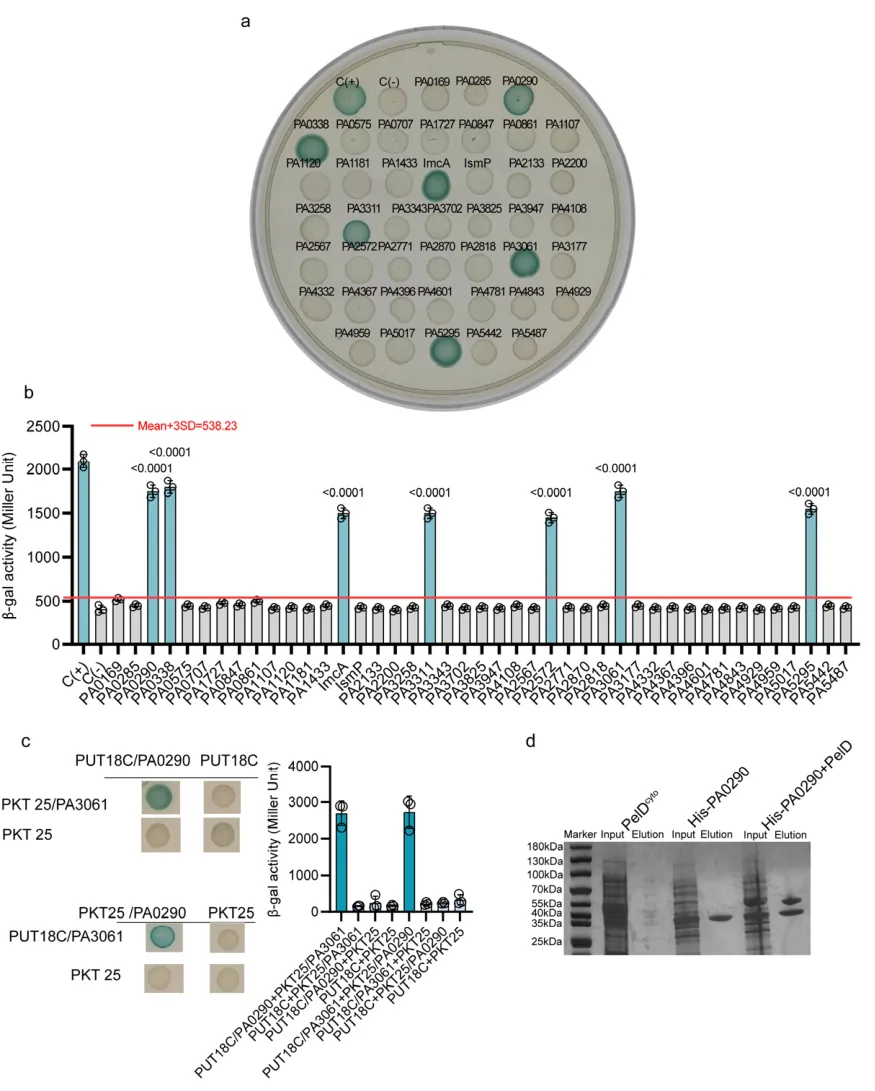
BACTH screening and affinity co-purification support an association between PA0290 and PelD. (**a**) Qualitative bacterial adenylate cyclase two-hybrid (BACTH) screening of candidate PA0290-interacting proteins. PA0290 was fused to the T18 fragment in pUT18C and tested against the indicated candidate proteins fused to the T25 fragment in pKT25. Co-transformants were cultured on LB agar supplemented with ampicillin, kanamycin, X-gal, and IPTG at 30 °C for 48 h. Blue colony development indicates reconstitution of adenylate cyclase activity and therefore a candidate protein–protein interaction. C(+) and C(−) denote the positive pUT18C-Zip/pKT25-Zip control and the empty-vector negative control, respectively. (**b**) Quantification of BACTH interaction signals by β-galactosidase activity, expressed in Miller units. The red horizontal line indicates the screening threshold, defined as the mean activity of the negative-control group plus three standard deviations (mean + 3 SD). Individual circles represent independent biological replicates, and bars show the mean ± SD (n = 3). Statistical comparisons were performed against the negative control using one-way ANOVA followed by Dunnett’s multiple-comparison test; adjusted *p* values are shown above the corresponding groups. (**c**) Reciprocal BACTH validation of the interaction signal between PA0290 and PelD (PA3061). PA0290 and PA3061 were fused to the T18 and T25 fragments in both orientations, and each fusion construct was also tested against the corresponding empty vector. Representative colony phenotypes are shown on the left, and β-galactosidase activities are shown on the right. Robust reporter activation was observed for both PA0290–PA3061 fusion orientations but not for the empty-vector controls. Individual circles represent independent biological replicates, and bars show the mean ± SD (n = 3). (**d**) Ni^2+^-affinity co-purification of His-tagged PA0290 with the untagged cytoplasmic region of PelD (PelD^cyto^). PelD^cyto^ and His-PA0290 were expressed individually or co-expressed in *E. coli*. Soluble lysates (Input) were subjected to Ni^2+^-NTA affinity purification, and the corresponding eluates (Elution) were analyzed by SDS-PAGE. A band consistent with the expected molecular mass of PelD^cyto^ was observed in the Ni^2+^–NTA elution fraction from the co-expression sample, whereas a corresponding band was not evident when PelD^cyto^ was expressed alone. These findings are consistent with an association between PA0290 and the cytoplasmic region of PelD under the tested conditions.

Using the mean activity of the negative control plus 3 SD as the screening threshold, 7 of the 43 candidates (16.3%) exceeded the cutoff (Table S3). These candidates included PA0290, PA0338, ImcA, PA3311, PA2572, PA3061 (PelD), and PA5295 (Figure 2a,b). Among these candidates, PelD generated a strong BACTH signal and was prioritized for further analysis because it is a well-characterized c-di-GMP receptor involved in Pel-dependent biofilm formation. Quantification of interaction strength by β-galactosidase activity further confirmed that the PA0290–PelD pair generated a significantly higher signal than the negative control and exceeded the predefined positive-interaction threshold (Figure 2b).

Reciprocal BACTH analysis further showed robust reporter activation when PA0290 and the PelD cytoplasmic region were tested in both T18/T25 fusion orientations, whereas the corresponding empty-vector controls remained at background levels (Figure 2c). To provide an independent biochemical assessment, His-tagged PA0290 was co-expressed with the untagged PelD cytoplasmic region and subjected to Ni^2+^-affinity purification. A band consistent with the expected molecular mass of PelD^cyto^ was observed in the Ni^2+^–NTA elution fraction from the His-PA0290/PelD^cyto^ co-expression sample, whereas a corresponding band was not evident when PelD^cyto^ was expressed alone under the same conditions (Figure 2d). Together, these results provide complementary evidence supporting an association between PA0290 and the cytoplasmic region of PelD.

PelD is a well-characterized c-di-GMP receptor involved in regulation of Pel exopolysaccharide synthesis and biofilm formation. Together, these observations suggested a potential functional relationship between PA0290 and PelD, which was examined further in subsequent biofilm assays.

### 3.3. PA0290 Cooperates with PelD to Promote Biofilm Formation in Both Static and Flow Conditions

**Figure 3:**
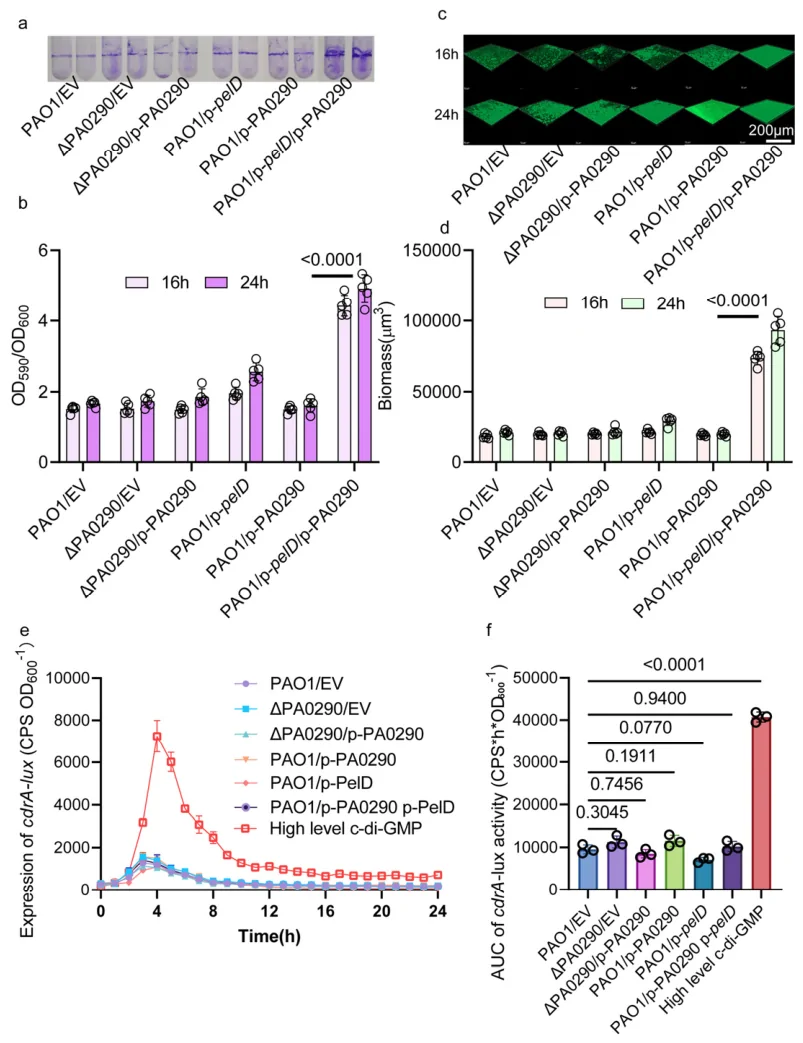
Co-expression of PA0290 and PelD enhances static and flow-cell biofilm formation without a sustained increase in bulk c-di-GMP reporter activity. (**a**) Representative crystal violet-stained static biofilms formed by PAO1/EV, ΔPA0290/EV, ΔPA0290/p-PA0290, PAO1/p-*pelD*, PAO1/p-PA0290, and the PA0290–PelD co-expression strain (PAO1/p-*pelD*/p-PA0290) after 16 and 24 h of incubation. (**b**) Quantification of static biofilm biomass. Crystal violet absorbance at 590 nm was normalized to bacterial growth at OD_600_. Co-expression of PA0290 and PelD markedly increased biofilm formation compared with expression of either protein alone. (**c**) Representative three-dimensional confocal laser scanning microscopy images of flow-cell biofilms formed by the indicated strains after 16 and 24 h. Biofilm cells were stained with SYTO 9. Scale bar, 200 μm. (**d**) Quantification of biofilm biomass from confocal image stacks. (**e**) Temporal activity of the c-di-GMP-responsive *cdrA-lux* reporter in the indicated strains over 24 h. The Δ*ismP*/p-*imcA* strain [11], which has an elevated global intracellular c-di-GMP level, was included as a high-c-di-GMP positive control. This control exhibited a pronounced increase in reporter activity, confirming the responsiveness and dynamic range of the reporter assay. In contrast, PAO1/EV, Δ*PA0290*/EV, Δ*PA0290*/p-PA0290, PAO1/p-PA0290, PAO1/p-*pelD*, and the PA0290–PelD co-expression strain displayed broadly comparable temporal profiles. Notably, PA0290–PelD co-expression did not produce a detectable sustained increase in bulk *cdrA-lux* reporter activity despite its marked enhancement of biofilm formation. Reporter activity is expressed as luminescence counts per second normalized to OD_600_ (CPS/OD_600_). (**f**) Quantification of cumulative *cdrA*-lux reporter activity by area-under-the-curve (AUC) analysis over the 0–24 h monitoring period. AUC values were calculated separately for each independent biological replicate. Individual symbols represent independent biological replicates, and bars indicate the mean ± SD (n = 3). Statistical comparisons were performed against PAO1/EV using one-way ANOVA followed by Dunnett’s multiple-comparison test; adjusted *p* values are shown. In (**b**,**d**,**f**), individual symbols represent independent biological replicates, and bars show the mean ± SD (n = 3). In (**e**), symbols and error bars represent the mean ± SD from three independent biological replicates.

To determine whether the PA0290–PelD interaction has functional consequences, we examined biofilm formation in strains expressing PA0290, PelD, or both proteins simultaneously. Expression of PA0290 alone or PelD alone produced only limited changes in biofilm biomass compared with the control strain. In contrast, co-expression of PA0290 and PelD resulted in a marked increase in static biofilm formation at both 16 h and 24 h (Figure 3a,b).

To further validate this phenotype under dynamic growth conditions, we established flow-cell biofilms and analyzed three-dimensional biofilm structures using confocal laser scanning microscopy. Consistent with the static biofilm assay, the PA0290–PelD co-expression strain displayed substantially increased biofilm biomass compared with all control strains after both 16 h and 24 h of cultivation (Figure 3c,d).

These results show that simultaneous expression of PA0290 and PelD produces a strong biofilm phenotype that was not observed when either protein was expressed alone under the same experimental conditions.

### 3.4. PA0290–PelD Co-Expression Is Not Accompanied by Sustained Activation of a Bulk c-di-GMP-Responsive Reporter

Because PA0290 contains a GGDEF domain harboring a GGEEF catalytic motif and markedly enhances biofilm formation when co-expressed with PelD, we next examined whether this phenotype was associated with a generalized increase in cellular c-di-GMP signaling. The c-di-GMP-responsive *cdrA-lux* reporter was introduced into the indicated strains to monitor population-level c-di-GMP-responsive transcriptional activity over 24 h. The Δ*ismP*/p-*imcA* strain, which exhibits an elevated global intracellular c-di-GMP level, was included as a high-c-di-GMP positive control.

The high-c-di-GMP positive control (Δ*ismP*/p-*imcA*) exhibited a pronounced increase in *cdrA*-lux activity, with a prominent peak at approximately 4 h followed by reporter activity that remained higher than that of the other strains throughout the monitoring period (Figure 3e). In contrast, PAO1/EV, ΔPA0290/EV, ΔPA0290/p-PA0290, PAO1/p-PA0290, PAO1/p-*pelD*, and the PA0290–PelD co-expression strain displayed broadly comparable temporal reporter profiles.

To formally compare cumulative reporter output, the 0–24 h AUC was calculated separately for each independent biological replicate. The high-c-di-GMP positive control showed a marked increase in cumulative *cdrA*-lux reporter activity compared with PAO1/EV (Dunnett-adjusted *p* < 0.0001), whereas no statistically significant increase was detected for the PA0290–PelD co-expression strain relative to PAO1/EV (adjusted *p* = 0.9400) (Figure 3f). No statistically significant increases were detected for the other tested strains under these conditions.

Thus, PA0290–PelD co-expression was not associated with a detectable sustained increase in bulk c-di-GMP-responsive reporter activity under the conditions tested. This observation is consistent with, but does not by itself demonstrate, a spatially restricted c-di-GMP signaling mechanism.

### 3.5. The Conserved GGEEF Motif Contributes to PA0290 Activity and PA0290–PelD-Associated Biofilm Enhancement

**Figure 4:**
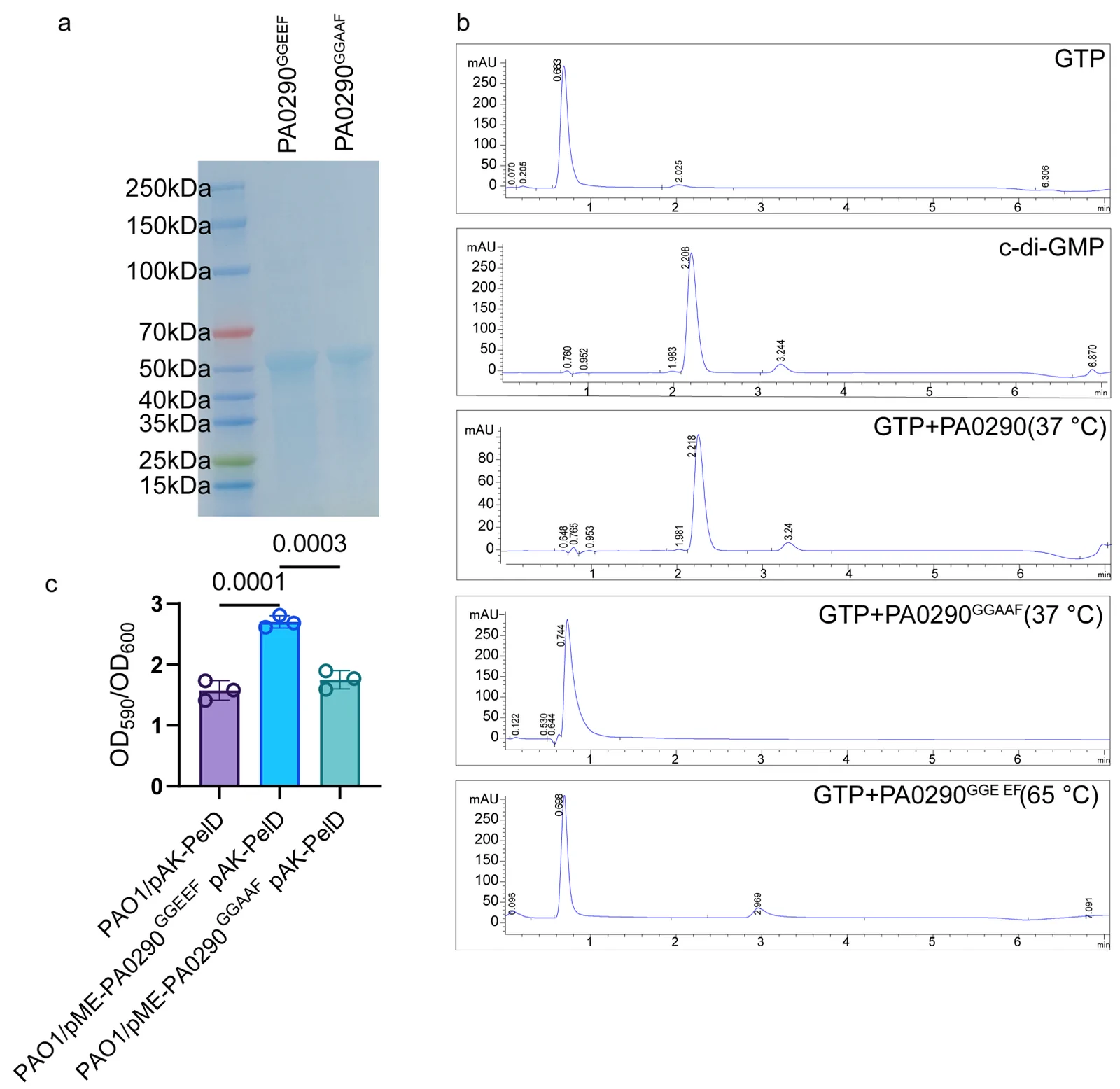
Mutation of the conserved GGEEF motif reduces PA0290 activity and PA0290–PelD-associated biofilm enhancement. (**a**) SDS-PAGE analysis of purified wild-type PA0290 containing the intact GGEEF motif and the PA0290^GGAAF^ variant. (**b**) Reverse-phase HPLC analysis of the in vitro enzymatic activity of PA0290. GTP and c-di-GMP standards were monitored at 254 nm and used to determine their respective retention positions. Reaction mixtures containing GTP and wild-type PA0290 produced a product peak with a retention time closely matching that of the authentic c-di-GMP standard. The corresponding product peak was absent or markedly reduced in the heat-inactivated PA0290 control and in the PA0290^GGAAF^ mutant reaction, supporting diguanylate cyclase activity of PA0290 and an important role of the GGEEF motif in product formation. (**c**) Static biofilm formation in strains expressing PelD alone, PelD together with wild-type PA0290, or PelD together with the PA0290^GGAAF^ mutant. Biofilm biomass was quantified by crystal violet staining and expressed as OD_590_/OD_600_. Co-expression of wild-type PA0290 with PelD increased biofilm formation, whereas the GGEEF-to-GGAAF substitution substantially reduced this effect. Individual circles represent independent biological replicates, and bars indicate the mean ± SD (n = 3).

To examine the contribution of the conserved GGEEF motif to PA0290 activity, we generated a PA0290^GGAAF^ variant in which the native GGEEF motif was replaced with GGAAF. Wild-type PA0290 and the PA0290GGAAF mutant were purified and analyzed by SDS-PAGE, confirming successful protein preparation (Figure 4a).

The enzymatic activity of PA0290 was examined using GTP as the substrate and reverse-phase HPLC analysis. A product peak with a retention time matching that of authentic c-di-GMP was detected in reactions containing wild-type PA0290 and GTP. The corresponding product peak was absent or markedly reduced in the heat-inactivated PA0290 control and in the PA0290^GGAAF^ reaction (Figure 4b). These results support diguanylate cyclase activity of PA0290 and indicate an important contribution of the conserved GGEEF motif to product formation.

We subsequently examined whether the GGEEF-to-GGAAF substitution affected the PA0290–PelD-associated biofilm phenotype. Whereas co-expression of wild-type PA0290 with PelD strongly promoted biofilm formation, the PA0290^GGAAF^ variant showed a substantially reduced ability to enhance biofilm formation when co-expressed with PelD (Figure 4c). Together, these results indicate that an intact GGEEF motif is important for the PA0290–PelD-associated biofilm phenotype.

### 3.6. Exploratory Analysis Reveals Positive Correlations Between Biofilm Formation and PA0290/pelD Expression in Clinical Isolates

**Figure 5:**
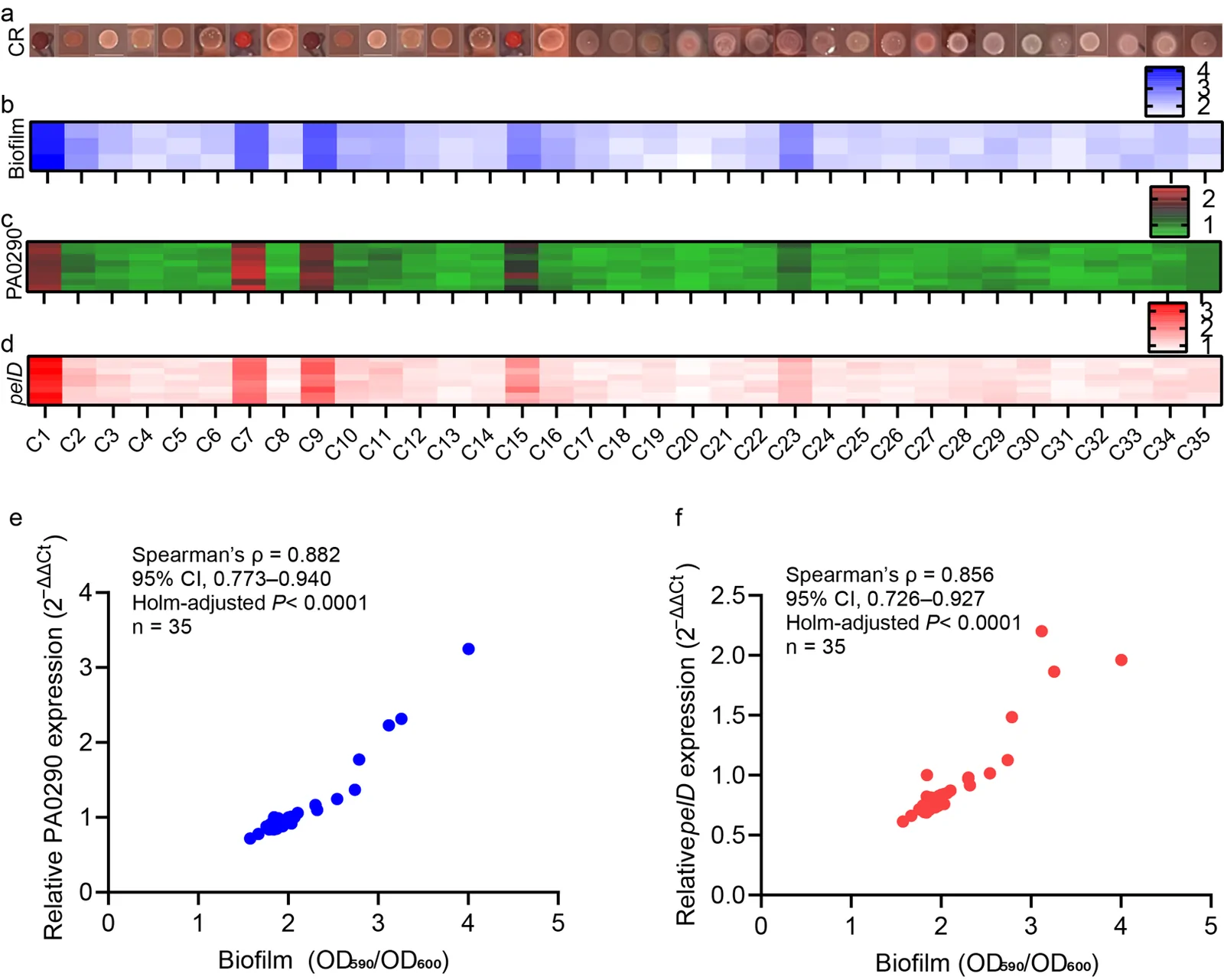
Biofilm heterogeneity and *PA0290*–*pelD* transcriptional variation among clinical *Pseudomonas aeruginosa* isolates. (**a**) Congo red colony morphologies of 35 clinical *P. aeruginosa* isolates. The isolates exhibited substantial variation in colony pigmentation and morphology, including red-pigmented, rugose, and small-colony-like phenotypes. (**b**) Heatmap showing the relative static biofilm-forming capacity of the 35 clinical isolates. Biofilm biomass was quantified by crystal violet staining, and color intensity represents the measured biofilm value according to the scale shown. (**c**) Heatmap showing relative *PA0290* mRNA abundance in the clinical isolates, as determined by quantitative reverse-transcription PCR. (**d**) Heatmap showing relative *pelD* mRNA abundance in the same isolates. Color intensity in panels c and d indicates relative transcriptional abundance according to the corresponding scales. (**e**,**f**) Scatterplots showing the relationships between static biofilm formation and relative *PA0290* (**e**) or *pelD* (**f**) transcript abundance across the 35 clinical isolates. Each point represents the mean value for one isolate. Associations were assessed using Spearman rank correlation. Ninety-five percent confidence intervals are shown for the correlation coefficients, and *p* values were adjusted for two comparisons using the Holm method.

To investigate whether the PA0290–PelD regulatory module contributes to clinically relevant phenotypic variation, we analyzed 35 non-duplicate clinical *P. aeruginosa* isolates. Congo red colony morphology analysis revealed substantial diversity among clinical isolates, including differences in pigmentation, colony architecture, and rugose-like morphology (Figure 5a).

Consistent with the colony phenotype variation, quantitative biofilm assays demonstrated considerable heterogeneity in biofilm-forming capacity among the clinical isolates (Figure 5b). We next examined transcriptional variation in PA0290 and *pelD* by quantitative reverse-transcription PCR. Both genes displayed substantial isolate-to-isolate variation in transcript abundance. Spearman correlation analysis revealed a strong positive correlation between biofilm formation and PA0290 transcript abundance (Spearman’s ρ = 0.882, 95% CI, 0.773–0.940, Holm-adjusted *p* < 0.0001; n = 35). Biofilm formation was also strongly positively correlated with *pelD* transcript abundance (Spearman’s ρ = 0.856, 95% CI, 0.726–0.927, Holm-adjusted *p* < 0.0001; n = 35) (Figure 5e,f).

Finally, phylogenetic analyses further showed that PA0290 and PelD-related proteins are broadly distributed across diverse bacterial taxa (Figure S1), indicating conservation of related protein families across multiple bacterial lineages.

## 4. Discussion

PA0290 was previously identified as a conserved GGDEF-domain protein with diguanylate cyclase activity in systematic analyses of the *Pseudomonas aeruginosa* c-di-GMP network [6,25]. Our study extends this assignment by linking PA0290 to the c-di-GMP receptor PelD and to a defined biofilm output. PA0290 deletion or overexpression alone produced little effect, whereas PA0290-PelD co-expression strongly increased static and flow-cell biofilms. This partner dependence is consistent with the conditional functions of individual *P. aeruginosa* diguanylate cyclases [7] and with other systems in which DGC activity is shaped by interaction partners, including DgcP-FimV and PilO-SadC [26,27]. PelD was therefore prioritized because it is a known c-di-GMP effector and generated a clear cooperative phenotype, although the additional BACTH-positive candidates suggest that PA0290 may participate in a broader interaction network.

The absence of a statistically detectable increase in cumulative *cdrA*-lux reporter activity, supported by 0–24 h AUC analysis, is important for interpreting this phenotype. Although *cdrA*-based reporters are widely used to monitor c-di-GMP-responsive transcription, they provide a population-averaged output rather than a direct measurement near a protein complex [28]. Local signaling is generally inferred when a pathway combines output specificity, physical association, and limited disturbance of the global c-di-GMP pool [8,9]. Protein-interaction hubs may further organize these pathway-specific outputs [29]. PA0290-PelD satisfies several elements of this framework, but spatially restricted nucleotide transfer has not yet been demonstrated. A recent RoeA study also showed that specific outputs can arise from diffusible c-di-GMP interpreted by a receptor hierarchy [30], reinforcing the need to distinguish local transfer from global receptor capture experimentally.

The PAS-PAC region of PA0290 suggests that an environmental cue may regulate this module. Binding partners can reorganize GGDEF enzymes into catalytically active assemblies, as shown for SiaC-SiaD [10]. The IsmP-ImcA system similarly links iron sensing to altered protein association, c-di-GMP synthesis, and biofilm behavior [11]. Other *P. aeruginosa* DGC pathways respond to peroxide or extracellular purines [12,13]. These examples indicate that distinct environmental inputs can be converted into selective c-di-GMP outputs. Identifying the ligand or physiological condition sensed by PA0290 will therefore be important for establishing when the PA0290-PelD pathway functions at native expression levels.

PelD is a biologically plausible output node because it is required for c-di-GMP-dependent Pel production [16] and contains a degenerate GGDEF-like receptor region [17]. It also forms part of the PelDEFG inner-membrane biosynthetic complex [31]. Pel contributes to matrix organization and can cross-link extracellular DNA; therefore, increased Pel production represents one biologically plausible mechanism by which PelD-associated signaling could enhance biofilm biomass [15,19,32]. Most importantly, BifA was recently shown to interact with PelD and regulate Pel-dependent biofilms without a corresponding stable increase in global c-di-GMP [20], providing a close precedent for enzyme-PelD coupling. Importantly, the present experiments do not establish that the biofilm phenotype produced by PA0290–PelD co-expression is mediated through increased Pel production. Experiments in a Pel-deficient background together with direct measurement of Pel production would be required to determine whether Pel is the downstream matrix component responsible for the observed co-expression phenotype.

Direct coupling can confer specificity by recruiting an enzyme to its target, enhancing catalytic activity, or protecting the output from cross-talk. In the pseudomonad Lap pathway, DGC-receptor interaction selectively controls adhesion, and ligand-enhanced association can stimulate biofilm formation [33,34]. DibA-LapD coupling provides a comparable PDE-effector example [35], while interaction-centered modules also operate in *Escherichia coli* and *Lysobacter enzymogenes* [36,37]. In *P. aeruginosa*, ProE-PqsE interaction extends this principle beyond matrix regulation to pyocyanin production [38]. By analogy, PelD may recruit PA0290 to the Pel machinery, capture newly synthesized c-di-GMP, or alter PA0290 activity. Validation in the native *P. aeruginosa* context, interface mapping, and biochemical reconstitution will be needed to distinguish these possibilities.

Previous systematic analysis also supports a condition-dependent role for PA0290. Eilers et al. reported that deletion of *PA0290* reduced cellular c-di-GMP levels and altered biofilm architecture, including impaired development of mushroom-like structures in PAO1 [39]. These findings are consistent with PA0290 functioning as an active diguanylate cyclase, while the absence of a statistically detectable phenotype in our static biofilm assay further suggests that its phenotypic output may depend on the experimental context and associated signaling partners.

Although the GGEEF-to-GGAAF substitution reduced both c-di-GMP product formation in vitro and the biofilm phenotype associated with PA0290–PelD co-expression, the present data do not exclude possible effects of this substitution on PA0290 stability, conformation, or interaction with PelD. Therefore, the mutant phenotype is interpreted here as evidence for the importance of the conserved GGEEF motif rather than definitive proof that loss of catalytic activity alone accounts for the observed biofilm phenotype.

The clinical-isolate analysis revealed strong positive correlations between biofilm formation and the transcript abundance of both *PA0290* and *pelD*. However, these associations should be interpreted cautiously. The isolates were designated as non-duplicate according to routine laboratory criteria but were not genotyped by MLST or whole-genome sequencing; therefore, potential lineage structure cannot be excluded as a confounding factor. In addition, correlation does not establish that variation in *PA0290* or *pelD* expression directly determines biofilm capacity in clinical isolates. Accordingly, these findings are considered exploratory support for the PAO1-based observations rather than direct validation of the PA0290–PelD regulatory model across clinical lineages.

## 5. Conclusions

In summary, our results support diguanylate cyclase activity of PA0290 and a functional association between PA0290 and PelD in biofilm regulation. Co-expression of PA0290 and PelD markedly enhanced biofilm formation, whereas disruption of the conserved GGEEF motif reduced this phenotype. These findings provide a basis for future studies addressing how PA0290–PelD association contributes to biofilm regulation.

## Data Availability

The original contributions presented in this study are included in the article/Supplementary Material. Further inquiries can be directed to the corresponding authors.

## Supplementary Materials

The following supporting information can be downloaded at: https://www.mdpi.com/article/10.3390/microorganisms14091961/s1, Table S1: Bacterial strains and plasmids used in this study; Table S2: Oligonucleotides used in this study; Table S3: Complete BACTH candidate panel and screening classification; Table S4: Substrate and product peak areas from HPLC analysis; Table S5: *PA0290* and *pelD* expression profiles in 35 clinical *Pseudomonas aeruginosa* isolates; Figure S1: Phylogenetic analysis of PA0290 and PelD homologues.
