# Supplementary material for: Co-Expression of PA0290 and PelD Enhances Pseudomonas aeruginosa Biofilm Formation
Source: Microorganisms. 2026 Sep 5;14(9):1961. doi: 10.3390/microorganisms14091961 (PMC13609476; doi:10.3390/microorganisms14091961)
Supplement: Supplementary file 1 [file microorganisms-14-01961-s001.zip › microorganisms-4513121-supplementary.pdf]

Supplementary Information

Co-expression of PA0290 and PelD enhances *Pseudomonas aeruginosa* biofilm formation

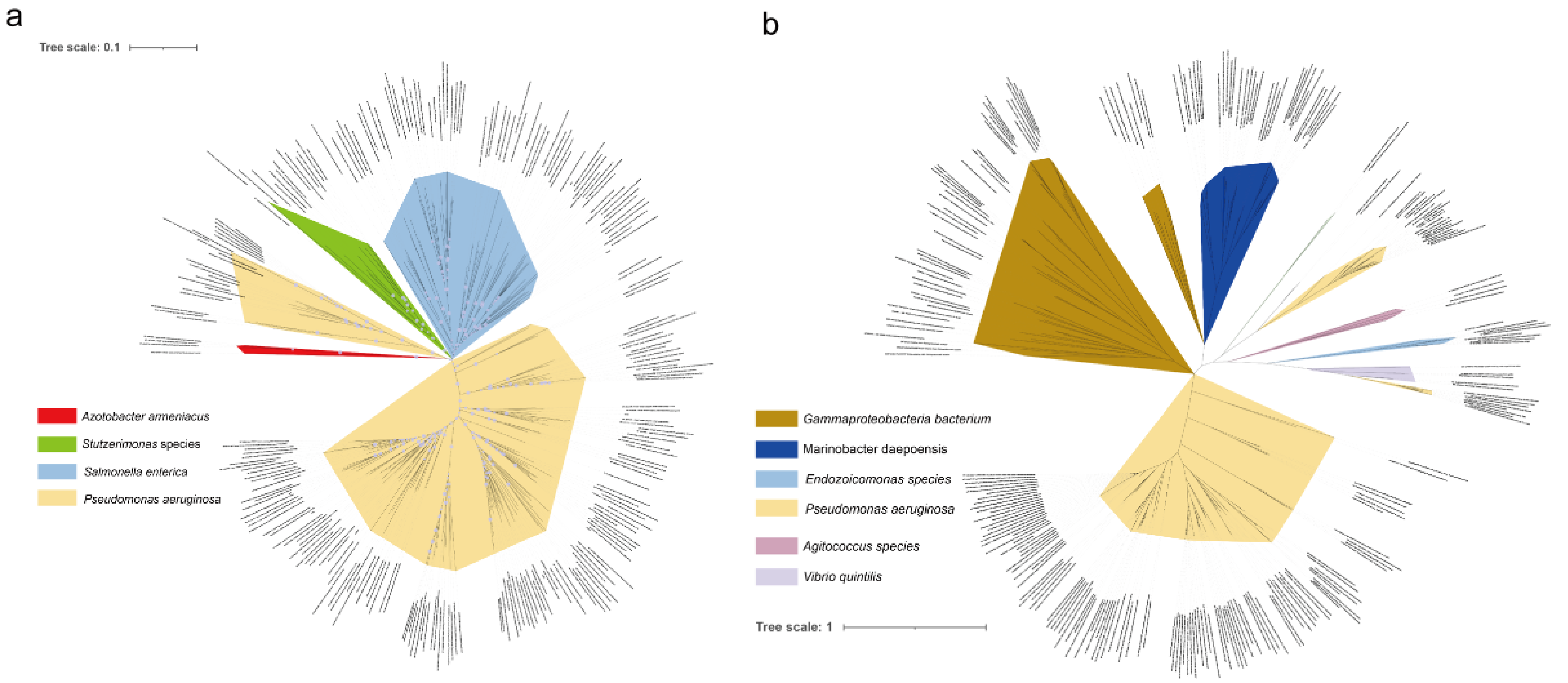

Supplementary Figure S1. Phylogenetic analysis of PA0290 and PelD homologues.
(a) Maximum-likelihood phylogenetic tree of PA0290 homologues. (b) Maximum-likelihood phylogenetic tree of PelD homologues. Colored sectors indicate representative bacterial taxa. Scale bars represent amino acid substitutions per site.

Supplementary Table S1. Bacterial strains and plasmids used in this study

Item	Type	Relevant genotype or construction	Selection/antibiotic concentration	Application in this study	Source or reference
Pseudomonas aeruginosa PAO1	Strain	Wild-type laboratory strain	TCS 25 µg/mL	Parental strain for genetic and phenotypic analyses	Laboratory collection
ΔPA0290	Strain	Markerless in-frame deletion of PA0290 in PAO1	TCS 25 µg/mL	Assessment of PA0290 function	This study
PAO1/pME6032	Strain	PAO1 carrying empty pME6032	TC 100 µg/mL	Matched empty-vector control for single-plasmid PA0290 assays	This study
ΔPA0290/pME6032	Strain	ΔPA0290 carrying empty pME6032	TC 100 µg/mL	Deletion-mutant empty-vector control	This study
ΔPA0290/pME-PA0290	Strain	ΔPA0290 carrying pME-PA0290	TC 100 µg/mL	Genetic complementation	This study
PAO1/pME-PA0290	Strain	PAO1 carrying pME-PA0290	TC 100 µg/mL	PA0290 overexpression	This study
PAO1/pME6032/pAK1900	Strain	PAO1 carrying empty pME6032 and empty pAK1900	TC 50 µg/mL + Carb 150 µg/mL	Matched dual-empty-vector control for co-expression assays	This study
ΔPA0290/pME6032/pAK190 0	Strain	ΔPA0290 carrying empty pME6032 and empty pAK1900	TC 50 µg/mL + Carb 150 µg/mL	Deletion-mutant control under dual-vector conditions	This study
ΔPA0290/pME-PA0290/pAK1900	Strain	ΔPA0290 carrying pME-PA0290 and empty pAK1900	TC 50 µg/mL + Carb 150 µg/mL	Complemented strain under dual-vector conditions	This study
PAO1/pME-PA0290/pAK1900	Strain	PAO1 carrying pME-PA0290 and empty pAK1900	TC 50 µg/mL + Carb 150 µg/mL	PA0290-only control under matched dual-vector conditions	This study
PAO1/pME6032/pAK-pelD	Strain	PAO1 carrying empty pME6032 and pAK-pelD	TC 50 µg/mL + Carb 150 µg/mL	PelD-only control under matched dual-vector conditions	This study
PAO1/pME-PA0290/pAK-pelD	Strain	PAO1 carrying pME-PA0290 and pAK-pelD	TC 50 µg/mL + Carb 150 µg/mL	PA0290–PelD co-expression biofilm assays	This study
PAO1/pME-PA0290GGAAF/pAK-pelD	Strain	PAO1 carrying pME-PA0290GGAAF and pAK-pelD	TC 50 µg/mL + Carb 150 µg/mL	Assessment of the GGEEF-to-GGAAF substitution in the PA0290–PelD co-expression phenotype	This study
Δ*ismP*/p-*imcA*	Strain	High-c-di-GMP positive-control strain carrying p-imcA in the ΔismP background	TC 50 µg/mL + Carb 150 µg/mL	Positive control for bulk c-di-GMP-responsive reporter activity	Ref. 11
Clinical *P. aeruginosa* isolates C1–C35	Strains	Thirty-five non-duplicate archived clinical isolates	TCS 25 µg/mL	Clinical biofilm and qRT-PCR analyses	Laboratory collection
Escherichia coli BTH101	Strain	Adenylate cyclase-deficient BACTH reporter strain	Amp 50 µg/mL + Kan 50 µg/mL	Bacterial two-hybrid assays	Laboratory collection
Escherichia coli BL21(DE3)	Strain	Protein-expression host	According to the expression construct	Expression of PA0290, PA0290GGAAF, and pETDuet co-purification constructs	Laboratory collection
pEX18Ap	Plasmid	Suicide vector for allelic exchange	Carb 300 µg/mL	Construction of ΔPA0290	Laboratory collection

Item	Type	Relevant genotype or construction	Selection/antibiotic concentration	Application in this study	Source or reference
pME6032	Plasmid	Compatible expression vector; empty-vector control	TC 100 µg/mL (single-vector); TC 50 µg/mL (dual-vector)	PA0290 expression and matched empty-vector control	Laboratory collection
pME-PA0290	Plasmid	PA0290 coding sequence cloned into pME6032	TC 100 µg/mL (single-vector); TC 50 µg/mL (dual-vector)	Complementation and PA0290 overexpression	This study
pME-PA0290GGAAF	Plasmid	PA0290 carrying the GGEEF-to-GGAAF substitution in pME6032	TC 50 µg/mL in dual-vector assays	GGEEF-motif variant analysis	This study
pAK1900	Plasmid	Compatible expression vector; empty-vector control	Carb 300 µg/mL (single-vector); Carb 150 µg/mL (dual-vector)	PelD expression and matched empty-vector control	Laboratory collection
pAK-pelD	Plasmid	pelD coding sequence cloned into pAK1900	Carb 300 µg/mL (single-vector); Carb 150 µg/mL (dual-vector)	PelD expression and PA0290–PelD co-expression assays	This study
pUT18C-PA0290	Plasmid	PA0290 fused to the T18 fragment	Amp 100 µg/mL	BACTH bait construct	This study
pKT25-candidate derivatives	Plasmids	Candidate c-di-GMP-related proteins fused to the T25 fragment	Kan 50 µg/mL	BACTH candidate screening	This study/laboratory collection
pKT25-PelDcyto	Plasmid	PelD cytoplasmic fragment fused to the T25 fragment; the same PelD fragment was used for affinity co-purification	Kan 50 µg/mL	Primary BACTH analysis of the PA0290–PelD association	This study
pKT25-PA0290	Plasmid	PA0290 fused to the T25 fragment	Kan 50 µg/mL	Reciprocal BACTH validation	This study
pUT18C-PelDcyto	Plasmid	PelD cytoplasmic fragment fused to the T18 fragment	Amp 100 µg/mL	Reciprocal BACTH validation	This study
pUT18C-Zip / pKT25-Zip	Plasmids	BACTH positive-control plasmid pair	Amp 50 µg/mL + Kan 50 µg/mL	Positive control for BACTH assays	Laboratory collection
pUT18C / pKT25	Plasmids	Empty BACTH vectors	Amp 50 µg/mL + Kan 50 µg/mL	Negative and fusion-vector controls for BACTH assays	Laboratory collection
cdrA-lux reporter plasmid	Plasmid	cdrA promoter fused to luxCDABE	Tmp 600 µg/mL	Bulk c-di-GMP-responsive reporter assay	Laboratory collection
pET-28a-SUMO-PA0290	Plasmid	Full-length wild-type PA0290 cloned into pET-28a-SUMO	Kan 50 µg/mL	PA0290 purification and in vitro DGC assay	This study
pET-28a-SUMO-PA0290GGAAF	Plasmid	Full-length PA0290GGAAF cloned into pET-28a-SUMO	Kan 50 µg/mL	PA0290GGAAF purification and in vitro DGC assay	This study
pETDuet-His-PA0290	Plasmid	pETDuet-based construct expressing His-tagged PA0290 alone	Amp 100 µg/mL	Single-protein control for Ni^{2+}–NTA affinity co-purification	This study
pETDuet-PelDcyto	Plasmid	pETDuet-based construct expressing untagged PelD cytoplasmic region alone	Amp 100 µg/mL	Single-protein control for Ni^{2+}–NTA affinity co-purification	This study

Item	Type	Relevant genotype or construction	Selection/antibiotic concentration	Application in this study	Source or reference
pETDuet-His-PA0290/PelDcyto	Plasmid	Dual-expression construct containing His-tagged PA0290 and untagged PelD cytoplasmic region in the two independent pETDuet expression cassettes	Amp 100 µg/mL	Affinity co-purification of PA0290 and the PelD cytoplasmic region	This study

Notes: For matched dual-vector comparisons, the corresponding empty vector was included whenever a gene insert was absent, and TC 50 µg/mL plus Carb 150 µg/mL was used across groups. Single-vector PA0290 assays used TC 100 µg/mL. The pETDuet constructs used for protein co-expression and affinity co-purification were selected with ampicillin (Amp, 100 µg/mL).

Supplementary Table S2. Oligonucleotides used in this study

Primer name	Sequence (5′–3′)	Purpose
PA0290-Up-F	CATGATTACGAATTCCGGTGTATTCGCCGGACGAC	Amplification of upstream homologous arm for PA0290 deletion
PA0290-Up-R	CCTCGTCATCTCTAGACTTCAGCCGGGCCACTTC	Amplification/fusion of upstream homologous arm
PA0290-Down-F	CCGGCTGAAGTCTAGAGATGACGAGGGACTCGGTGC	Amplification/fusion of downstream homologous arm
PA0290-Down-R	GACTCTAGAGGATCCGCCAGTTGTGGATCAGGCCG	Amplification of downstream homologous arm
PA0290-Comp-F	attgaattcATGGACGACCTAACCGGCATTCCC	Cloning of intact PA0290 for complementation/overexpression
PA0290-Comp-R	attctcgagTCAGCCCACGACGATGCAGTCC	Cloning of intact PA0290 for complementation/overexpression
PA0290-GGAAF-F	GctGctTTCGCCGTGCTGC	Site-directed mutagenesis of GGEEF to GGAAF
PA0290-GGAAF-R	CACGGCGAAagCagCGCCGCCGATCCGCCCGAAC	Site-directed mutagenesis of GGEEF to GGAAF
pelD-F	TAGAATACTCAAGCTTATGTCCGCGCACAAGGATTTC	*Cloning of pelD into pAK1900*
pelD-R	tctagaAATAGCCACTTGCTGATCATTCAGGCCG	*Cloning of pelD into pAK1900*
PA0290-qF	TAACCGGCATTCCCGAAGAC	qRT-PCR of PA0290
PA0290-qR	ACGGCCGAAGATTTTCTCGT	qRT-PCR of PA0290
pelD-qF	ACAAGGATTTCACCCTCGCA	*qRT-PCR of pelD*
pelD-qR	GGATCACCCAGGGAAAGGTG	*qRT-PCR of pelD*
rpoD-qF	CGCAACAGCAATCTCGTCTGAAA	qRT-PCR internal reference
rpoD-qR	GCGGATGATGTCTTCCACCTGTT	qRT-PCR internal reference
PA0290-BTH-F	CGACTCTAGAGGATCCCATGGACGACCTAACCGGC	Construction of PA0290 BACTH fusion
PA0290-BTH-R	CGATGAATTCGAGCTCGGTCAGCCCACGACGATGCAGTCC	Construction of PA0290 BACTH fusion
PA0290-Protein-F	ATTGGATCCATGGACGACCTAACCGGC	Construction of His-sumo-tag PA0290 expression plasmid
PA0290-Protein-R	ATTAAGCTTTCAGCCCACGACGATGCAG	Construction of His-sumo-tag PA0290 expression plasmid

Supplementary Table S3. Complete BACTH candidate panel and screening classification

Candidate	β-galactosidase activity (mean ± SD, Miller units)	Fold vs C(-)	Cutoff	Classification
PA0169	528.0 ± 47.1	1.28	538.23	Negative
PA0285	475.3 ± 70.2	1.15	538.23	Negative
PA0290	1824.1 ± 289.5	4.41	538.23	**Positive**

PA0338	1750.5 ± 121.0	4.24	538.23	**Positive**
PA0575	427.8 ± 14.0	1.04	538.23	Negative
PA0707	433.3 ± 48.8	1.05	538.23	Negative
PA1727	487.0 ± 38.5	1.18	538.23	Negative
PA0847	488.2 ± 50.8	1.18	538.23	Negative
PA0861	473.4 ± 44.1	1.15	538.23	Negative
PA1107	442.0 ± 29.3	1.07	538.23	Negative
PA1120	443.7 ± 67.6	1.07	538.23	Negative
PA1181	449.9 ± 72.9	1.09	538.23	Negative
PA1433	448.8 ± 12.9	1.09	538.23	Negative
ImcA	1509.5 ± 71.4	3.65	538.23	**Positive**
IsmP	458.9 ± 52.8	1.11	538.23	Negative
PA2133	433.0 ± 50.2	1.05	538.23	Negative
PA2200	449.8 ± 53.3	1.09	538.23	Negative
PA3258	456.9 ± 56.2	1.11	538.23	Negative
PA3311	1575.9 ± 153.2	3.81	538.23	**Positive**
PA3343	480.5 ± 76.8	1.16	538.23	Negative
PA3702	459.3 ± 15.0	1.11	538.23	Negative
PA3825	461.1 ± 19.8	1.12	538.23	Negative
PA3947	448.2 ± 68.1	1.08	538.23	Negative
PA4108	493.1 ± 33.3	1.19	538.23	Negative
PA2567	425.0 ± 44.5	1.03	538.23	Negative
PA2572	1617.9 ± 220.0	3.91	538.23	**Positive**
PA2771	445.0 ± 27.6	1.08	538.23	Negative
PA2870	440.9 ± 48.1	1.07	538.23	Negative
PA2818	469.7 ± 41.3	1.14	538.23	Negative
PA3061	1961.0 ± 170.3	4.74	538.23	**Positive**
PA3177	458.5 ± 34.0	1.11	538.23	Negative
PA4332	425.5 ± 62.0	1.03	538.23	Negative
PA4367	426.7 ± 45.2	1.03	538.23	Negative
PA4396	466.7 ± 54.0	1.13	538.23	Negative
PA4601	427.8 ± 49.4	1.03	538.23	Negative

PA4781	448.3 ± 38.6	1.08	538.23	Negative
PA4843	445.8 ± 36.2	1.08	538.23	Negative
PA4929	407.9 ± 36.6	0.99	538.23	Negative
PA4959	446.5 ± 48.6	1.08	538.23	Negative
PA5017	469.2 ± 57.2	1.14	538.23	Negative
PA5295	1438.2 ± 36.6	3.48	538.23	**Positive**
PA5442	491.8 ± 70.0	1.19	538.23	Negative
PA5487	491.4 ± 58.5	1.19	538.23	Negative

Note: The screening cutoff is defined as the mean β-galactosidase activity of the C (−) control plus three standard deviations (mean + 3 SD). With the supplied C (−) values 371.684, 413.310, and 454.971 Miller units, the cutoff is 538.229 Miller units (≈538.23). C (+) and C (−) are controls and are not included in the 43-candidate hit-rate denominator.

Supplementary Table S4. Substrate and product peak areas from HPLC analysis

Condition	GTP retention time (min)	GTP peak area (mAU·min)	Product retention time (min)	Product peak area (mAU·min)	n (technical replicates)
GTP standard	0.688 ± 0.003	42.6 ± 0.8	—	0.00	3
c-di-GMP standard	—	0.00	2.208 ± 0.003	39.8 ± 0.7	3
GTP + PA0290 WT (37 °C)	0.703 ± 0.004	2.37 ± 0.25	2.218 ± 0.003	14.6 ± 0.6	3
GTP + PA0290GGAAF (37 °C)	0.744 ± 0.004	40.4 ± 0.7	ND	0.32 ± 0.03	3
GTP + heat-inactivated PA0290 (65 °C)	0.698 ± 0.003	41.6 ± 0.7	ND	0.24 ± 0.03	3

Supplementary Table S5. *PA0290* and *pelD* expression profiles in 35 clinical *Pseudomonas aeruginosa* isolates

Isolate	Biofilm OD590/OD600 (mean ± SD)	*rpoD* Ct (mean ± SD)	*PA0290* Ct (mean ± SD)	*PA0290* ΔCt (mean)	*PA0290* expression (mean ± SD)	*pelD* Ct (mean ± SD)	*pelD* ΔCt (mean)	*pelD* expression (mean ± SD)
C1	4.004 ± 0.171	18.568 ± 0.121	23.019 ± 0.212	4.451	3.300 ± 0.400	24.655 ± 0.182	6.086	1.261 ± 0.119
C2	2.544 ± 0.140	18.495 ± 0.130	23.351 ± 0.230	4.856	2.492 ± 0.327	24.247 ± 0.252	5.752	1.590 ± 0.238
C3	2.104 ± 0.130	18.487 ± 0.104	24.052 ± 0.155	5.565	1.524 ± 0.122	24.305 ± 0.187	5.819	1.518 ± 0.164

C4	1.836 ± 0.104	18.659 ± 0.108	25.578 ± 0.152	6.918	0.596 ± 0.044	25.786 ± 0.211	7.127	0.613 ± 0.077
C5	1.916 ± 0.115	18.088 ± 0.077	23.755 ± 0.227	5.666	1.421 ± 0.211	24.980 ± 0.172	6.892	0.722 ± 0.077
C6	1.973 ± 0.166	18.627 ± 0.150	25.342 ± 0.224	6.715	0.687 ± 0.079	24.513 ± 0.256	5.886	1.449 ± 0.209
C7	3.118 ± 0.008	18.938 ± 0.070	23.692 ± 0.171	4.754	2.673 ± 0.289	24.221 ± 0.186	5.283	2.200 ± 0.263
C8	1.872 ± 0.130	18.749 ± 0.121	24.990 ± 0.187	6.241	0.954 ± 0.094	25.582 ± 0.254	6.833	0.752 ± 0.117
C9	3.258 ± 0.042	18.231 ± 0.138	22.885 ± 0.176	4.653	2.868 ± 0.217	23.716 ± 0.174	5.484	1.914 ± 0.141
C10	2.301 ± 0.092	18.168 ± 0.164	24.037 ± 0.264	5.869	1.235 ± 0.177	24.455 ± 0.195	6.287	1.097 ± 0.081
C11	2.304 ± 0.031	17.894 ± 0.072	23.053 ± 0.156	5.160	2.018 ± 0.193	23.512 ± 0.149	5.618	1.745 ± 0.157
C12	2.002 ± 0.029	18.742 ± 0.164	24.510 ± 0.272	5.768	1.324 ± 0.199	25.987 ± 0.208	7.245	0.565 ± 0.050
C13	1.837 ± 0.085	18.839 ± 0.131	25.419 ± 0.242	6.580	0.754 ± 0.106	25.319 ± 0.181	6.480	0.960 ± 0.083
C14	1.906 ± 0.011	18.099 ± 0.134	24.408 ± 0.180	6.309	0.910 ± 0.076	24.873 ± 0.230	6.774	0.783 ± 0.101
C15	2.787 ± 0.101	18.325 ± 0.169	23.282 ± 0.220	4.957	2.323 ± 0.228	23.675 ± 0.212	5.350	2.100 ± 0.186
C16	2.321 ± 0.088	18.614 ± 0.158	23.166 ± 0.183	4.552	3.076 ± 0.195	24.031 ± 0.208	5.417	2.005 ± 0.187
C17	2.036 ± 0.039	18.284 ± 0.151	23.545 ± 0.231	5.261	1.882 ± 0.227	24.303 ± 0.226	6.020	1.321 ± 0.154
C18	1.788 ± 0.120	18.224 ± 0.149	24.871 ± 0.195	6.647	0.720 ± 0.063	25.174 ± 0.240	6.950	0.693 ± 0.090
C19	1.797 ± 0.184	18.457 ± 0.129	25.240 ± 0.164	6.783	0.655 ± 0.046	25.878 ± 0.196	7.421	0.500 ± 0.051
C20	1.576 ± 0.137	18.924 ± 0.152	26.181 ± 0.227	7.257	0.472 ± 0.055	26.286 ± 0.222	7.362	0.521 ± 0.058
C21	1.758 ± 0.107	18.168 ± 0.146	25.019 ± 0.243	6.851	0.625 ± 0.084	25.472 ± 0.208	7.303	0.542 ± 0.056
C22	1.952 ± 0.038	18.397 ± 0.126	25.518 ± 0.238	7.122	0.518 ± 0.072	24.550 ± 0.160	6.153	1.204 ± 0.082

C23	2.741 ± 0.092	18.162 ± 0.104	23.220 ± 0.141	5.058	2.165 ± 0.144	23.713 ± 0.169	5.551	1.827 ± 0.169
C24	1.870 ± 0.093	18.323 ± 0.122	25.377 ± 0.154	7.054	0.543 ± 0.035	25.391 ± 0.216	7.068	0.639 ± 0.079
C25	1.931 ± 0.065	18.387 ± 0.160	24.357 ± 0.191	5.970	1.151 ± 0.083	24.741 ± 0.225	6.354	1.047 ± 0.115
C26	1.668 ± 0.346	18.700 ± 0.119	26.025 ± 0.211	7.325	0.450 ± 0.055	25.885 ± 0.151	7.186	0.589 ± 0.038
C27	1.880 ± 0.080	18.437 ± 0.075	25.627 ± 0.180	7.189	0.494 ± 0.056	24.976 ± 0.162	6.539	0.922 ± 0.092
C28	1.841 ± 0.132	18.647 ± 0.084	25.159 ± 0.220	6.512	0.791 ± 0.111	25.303 ± 0.153	6.656	0.850 ± 0.076
C29	2.033 ± 0.094	18.337 ± 0.095	23.699 ± 0.140	5.362	1.754 ± 0.125	24.934 ± 0.218	6.597	0.885 ± 0.120
C30	1.886 ± 0.122	18.554 ± 0.160	24.998 ± 0.246	6.444	0.829 ± 0.108	25.269 ± 0.190	6.715	0.816 ± 0.059
C31	1.668 ± 0.084	18.628 ± 0.091	25.614 ± 0.183	6.986	0.569 ± 0.063	25.637 ± 0.177	7.009	0.665 ± 0.070
C32	1.937 ± 0.077	18.985 ± 0.109	25.361 ± 0.245	6.376	0.869 ± 0.132	25.205 ± 0.189	6.220	1.149 ± 0.124
C33	1.969 ± 0.170	18.024 ± 0.130	24.096 ± 0.181	6.072	1.073 ± 0.093	23.977 ± 0.161	5.953	1.384 ± 0.091
C34	2.067 ± 0.063	17.945 ± 0.079	23.409 ± 0.186	5.464	1.635 ± 0.191	23.630 ± 0.240	5.685	1.666 ± 0.262
C35	1.843 ± 0.145	18.303 ± 0.137	24.476 ± 0.185	6.173	1.000 ± 0.086	24.724 ± 0.269	6.421	1.000 ± 0.160
